# Comparative study of peritoneal dialysis versus hemodialysis on the clinical outcomes in Korea: a population-based approach

**DOI:** 10.1038/s41598-019-42508-z

**Published:** 2019-04-11

**Authors:** Sung Woo Lee, Na Rae Lee, Soo Kyung Son, Jimin Kim, Ah Ram Sul, Yunjung Kim, Jung Tak Park, Jung Pyo Lee, Dong-Ryeol Ryu

**Affiliations:** 1 Department of Nephrology, , Internal Medicine, Nowon Eulji Medical center, Eulji University, Seoul, Korea; 2 National Evidence-based Healthcare Collaborating Agency, Seoul, Korea; 3 Department of Internal Medicine, College of Medicine, Yonsei University, Seoul, Korea; 4 Department of Internal Medicine, Seoul National University Boramae Medcal Center, Seoul, Korea; 5 Department of Internal Medicine, Seoul National University College of Medicine, Seoul, Korea; 6 Department of Internal Medicine, School of Medicine, Ewha Womans University, Seoul, Korea

## Abstract

There has been paucity of data regarding the secular trend of adverse outcomes in peritoneal dialysis (PD) as compared with hemodialysis (HD) in Korea. 96,596 patients who started dialysis between 2004–2015 in Korea were identified using the National Health Insurance Service database. The adjusted hazard ratio (HR) (95% confidence interval, CI) of PD over HD for mortality was 1.31 (1.27–1.36; *P* < 0.001) in the period of 2004–2007 and 1.21 (1.16–1.27; *P* < 0.001) in the period of 2008–2011. However, the hazard of PD over HD for mortality turned out to be insignificant in the period of 2012–2015. Similar trend was noted for nonfatal cardiovascular events (CVEs). In subgroup analysis, the hazard of PD over HD for mortality was evident, regardless of the status of age, diabetes, and comorbidity burden in 2004–2011. In 2012–2015, however, the hazard of PD over HD for mortality was insignificant when follow up was censored at one year, which became significant when follow up follow up was censored at three or five year. In conclusion, the mortality of PD over HD in Korea has been significantly improved, a finding that was paralleled by the improved nonfatal CVEs.

## Introduction

In Korea, the overall number of patients with end-stage renal disease (ESRD) reached 80,674 at the end of 2014^1^. Among ESRD patients, 70% were on hemodialysis (HD) and 19.8% had undergone kidney transplantation. However, only 9.2% were on peritoneal dialysis (PD), which was attributed to the net decrease in the prevalence of PD patients since the mid-2000s^1^. The decrease of PD patients is not a unique feature of Korea. In the United States, the incidence of PD has decreased since the mid-1980s, while the prevalence of PD has decreased since the mid-1990s^2^. The exact reason for why the PD prevalence in Korea has decreased is not known at this time. However, the increased age of ESRD patients in Korea^3^ may have a large effect because increased age is the independent determinant in the choice to undergo HD^4^. In addition, a lack of PD-related infrastructures, inadequate predialysis patient education, and insufficient physician training may also have had an influence on the decrease of PD^5^.

As compared with HD, PD has several advantages. First, PD is less expensive than HD^6^. Second, more patients with PD maintain their jobs compared with those using HD because of the increased schedule flexibility^7^. Third, patients with PD showed higher treatment satisfaction than did those with HD^8^. Fourth, patients with PD may have more favorable transplantation outcomes than those with HD^9^. Despite these advantages, however, efforts to revive PD have fallen into insignificance because of the lack of confidence in survival benefits of PD over HD. Our group recently reported that the survival rate associated with PD was inferior to that with HD among Korean ESRD patients who initiated dialysis between 2005 and 2008, using data from the national insurance database^10^. However, the outcomes of PD have been improved worldwide as time has gone by^11–14^, and Korea seems to be no exception^15,16^. Therefore, we performed the current study using a nationwide retrospective cohort to identify whether the survival rate with PD has indeed been improved and to explore the secular trends of other important clinical outcomes of PD in Korea.

## Results

We explored the baseline characteristics of the study population according to the three study periods of 2004 to 2007, 2008 to 2011, and 2012 to 2015 (Table 1). Regardless of the study period considered, HD patients were older than PD patients, but the difference in age was further increased with the progression of study period. The proportion of medical aid was higher in HD patients than in PD patients, while income status was lower in HD patients than in PD patients, but this difference decreased in magnitude as the study period increased. In the period of 2004 to 2007, more PD patients were diabetic, but the proportion of diabetes in HD patients exceeded that in PD patients thereafter. In the period of 2004 to 2007, PD patients showed similar previous stroke and higher previous coronary artery disease (CAD) rates, respectively, but the rates of previous stroke and CAD seemed to be increased only in HD patients thereafter. Although the rate of atrial fibrillation was similar in PD patients throughout the study period, it was increased in HD patients with the progression of the study period. More PD patients than HD patients used statin throughout the study period, whereas more HD patients than PD patients used antiplatelet agents and anticoagulants throughout the study period.

**Table 1:** Baseline characteristics of the study population according to the study period

	2004–2007 (n = 32,794)			2008–2011 (n = 30,518)			2012–2015 (n = 33,329)		
	PD (n = 7,553)	HD (n = 25,241)	*P*	PD (n = 5,968)	HD (n = 24,550)	*P*	PD (n = 4,699)	HD (n = 28,630)	*P*
Age (years)	53.9 ± 13.6	55.7 ± 13.8	<0.001	54.2 ± 14.0	60.2 ± 14.0	<0.001	54.6 ± 14.1	62.5 ± 13.9	<0.001
Male sex (n, %)	4,198 (55.6)	14,649 (58.0)	<0.001	3,496 (58.6)	14,511 (59.1)	0.46	2,763 (58.8)	17,285 (60.4)	0.041
Medical aid (n, %)	1,430 (18.9)	8,418 (33.4)	<0.001	583 (9.8)	3,470 (14.1)	<0.001	321 (6.8)	2,764 (9.7)	<0.001
Income status	8.4 ± 7.1	7.0 ± 7.3	<0.001	10.3 ± 6.6	9.9 ± 7.0	<0.001	10.4 ± 6.5	10.4 ± 6.8	0.9
Diabetes (n, %)	4,093 (54.2)	11,488 (45.5)	<0.001	3,486 (58.4)	1,5249 (62.1)	<0.001	2,791 (59.4)	18,072 (63.1)	<0.001
Hypertension (n, %)	7,344 (97.2)	19,593 (77.6)	<0.001	5,915 (99.1)	2,4011 (97.8)	<0.001	4,637 (98.7)	27,966 (97.7)	<0.001
Previous stroke (n, %)	786 (10.4)	2,514 (10.0)	0.26	701 (11.8)	4,022 (16.4)	<0.001	498 (10.6)	4,733 (16.5)	<0.001
Previous CAD (n, %)	786 (10.4)	1,750 (6.9)	<0.001	585 (9.8)	2,475 (10.1)	0.52	426 (9.1)	3,021 (10.6)	0.002
Malignancy (n, %)	196 (2.6)	1,340 (5.3)	<0.001	195 (3.3)	1,968 (8.0)	<0.001	198 (4.2)	2,610 (9.1)	<0.001
Liver disease (n, %)	813 (10.76)	2,711 (10.74)	0.9	744 (12.5)	3,366 (13.7)	0.01	559 (11.9)	3,836 (13.4)	0.005
Lung disease (n, %)	665 (8.8)	2,078 (8.2)	0.12	697 (11.7)	3,204 (13.1)	0.004	562 (12.0)	4,323 (15.1)	<0.001
Atrial fibrillation (n, %)	102 (1.4)	239 (1.0)	0.002	87 (1.5)	455 (1.9)	0.04	78 (1.7)	707 (2.5)	0.001
Hypothyroidism (n, %)	168 (2.2)	405 (1.6)	<0.001	181 (3.0)	764 (3.1)	0.75	196 (4.2)	1,089 (3.8)	0.23
CCI score									
1–3	3,506 (46.4)	13,477 (53.4)	<0.001	2,366 (39.6)	7,891 (32.1)	<0.001	1,929 (41.1)	9,485 (33.1)	<0.001
4–6	3,545 (46.9)	9,993 (39.6)	<0.001	3,066 (51.4)	13,390 (54.5)	<0.001	2,358 (50.2)	15,330 (53.6)	<0.001
≥7	502 (6.7)	1,771 (7.0)	<0.001	536 (9.0)	3,269 (13.3)	<0.001	412 (8.8)	3,815 (13.3)	<0.001
ACEI/ARB (n, %)	7,186 (95.1)	22,500 (89.1)	<0.001	5,649 (94.7)	22,345 (91.0)	<0.001	4,173 (88.8)	23,958 (83.7)	<0.001
Other anti-HTN drugs (n, %)	7,500 (99.3)	24,424 (96.7)	<0.001	5,933 (99.4)	24,179 (98.5)	<0.001	4,610 (98.1)	27,669 (96.6)	<0.001
OAD (n, %)	3,183 (42.1)	9,576 (37.9)	<0.001	2,470 (41.4)	10,270 (41.8)	0.53	2,007 (42.7)	12,402 (43.3)	0.44
Statin (n, %)	5,256 (69.6)	13,395 (53.1)	<0.001	4,367 (73.2)	14,385 (58.6)	<0.001	3,089 (65.7)	16,039 (56.0)	<0.001
Anti-platelet agents (n, %)	5,894 (78.0)	21,985 (87.1)	<0.001	4,316 (72.3)	21,126 (86.1)	<0.001	2,455 (52.3)	21,435 (74.9)	<0.001
Anti-coagulants (n, %)	615 (8.1)	2,524 (10.0)	<0.001	400 (6.7)	2,214 (9.0)	<0.001	193 (4.1)	1,897 (6.6)	<0.001

PD, peritoneal dialysis; HD, hemodialysis; CAD, coronary artery disease; ACEI/ARB, angiotensinogen-converting enzyme inhibitor/angiotensin receptor blocker; HTN, hypertension; OAD, oral antidiabetic drug; ESA, erythropoiesis-stimulating agent; CCI, Charlson Comorbidity Index. Values are expressed as the mean ± standard deviation for continuous variables and n (%) for categorical variables. Differences were evaluated by t-tests for continuous variables and chi-squared tests for categorical variables.

**Figure 1:**
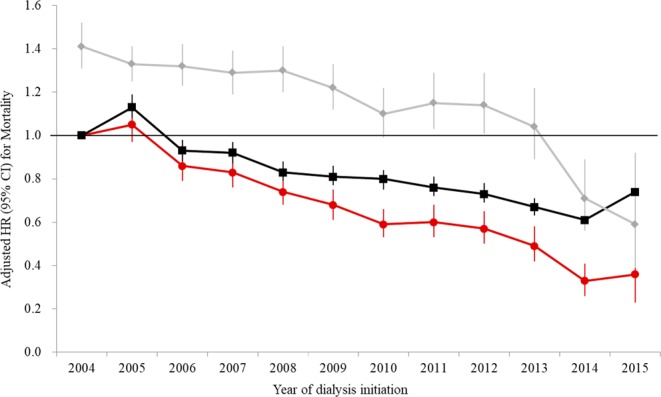
Secular trend of mortality according to dialysis modality and the respective hazard of PD over HD. The black line indicates mortality trends of HD, while the red line indicates that of PD according to the year of dialysis initiation. The gray line indicates the respective hazard ratio of PD over HD. Adjusted HRs and 95% CIs were calculated by multivariate Cox proportional hazard regression analysis, entering age, sex, insurance type, income status, and CCI value as covariates. HR, hazard ratio; CI, confidence interval; HD, hemodialysis; PD, peritoneal dialysis.

We explored the crude mortality rate according to dialysis modality (Table 2). In the period of 2004 to 2007, the mortality rate of PD patients (105.8 per 1,000 person-years) was higher than that of HD patients (89.1 per 1,000 person-years), which was confirmed by the finding that the hazard ratio (HR) (95% confidence interval, CI) of PD over HD for mortality was 1.31 (1.27–1.36; *P* < 0.001) in multivariate Cox proportional hazard regression analysis (Table 3). However, the mortality of PD seemed to be improved thereafter, as the hazard of PD over HD for mortality was not statistically significant in the period of 2012 to 2015 (HR: 1.00, 95% CI: 0.91–1.09; *P* = 0.961). We further analyzed mortality trends of dialysis modality and the respective hazard of PD over HD according to the year of dialysis initiation. In comparison with 2004, the hazard of mortality was significantly decreased in both PD and HD patients. However, the improvement was much higher in PD than in HD patients, which resulted in the net decrease of the hazard of mortality of PD over HD as the study period progressed and, ultimately, the mortality of PD presented as statistically lower than that of HD beginning in 2014 (Fig. 1).

**Table 2:** Crude incidence rates of major clinical outcomes of dialysis modality according to the study period.

	2004–2007 (n = 32,794)				2008–2011 (n = 30,518)				2012–2015 (n = 33,329)			
	PD (n = 7,553)		HD (n = 25,241)		PD (n = 5,968)		HD (n = 24,550)		PD (n = 4,699)		HD (n = 28,630)	
	N (%)	1,000 PY	N (%)	1 000 PY	N (%)	1,000 PY	N (%)	1,000 PY	N (%)	1,000 PY	N (%)	1,000 PY
Mortality	4,326 (57.3)	105.8	13,785 (54.6)	89.1	2,215 (37.1)	87.6	10,475 (42.7)	103.7	582 (12.4)	66	5,285 (18.5)	105.3
Nonfatal CVE	1,380 (18.3)	37.3	4,653 (18.4)	33.1	842 (14.1)	35.8	3,827 (15.6)	41.5	334 (7.1)	39.6	2,696 (9.4)	57.4
AMI	414 (5.5)	10.4	1,267 (5.0)	8.4	219 (3.7)	8.8	919 (3.7)	6.2	116 (1.9)	4.2	587 (2.4)	5.6
Any stroke	1,049 (13.9)	27.7	3,657 (14.5)	25.5	657 (11.0)	27.5	3,068 (12.5)	32.7	230 (4.9)	26.9	2,114 (7.4)	44.4
Ischemic stroke	242 (3.2)	6	944 (3.7)	6.2	134 (2.2)	5.4	711 (2.9)	7.2	58 (1.2)	6.6	467 (1.6)	9.4
Hemorrhagic stroke	717 (9.5)	18.4	2480 (9.8)	16.9	440 (7.4)	18.1	2,005 (8.2)	20.9	136 (2.9)	15.7	1,373 (4.8)	28.3

PD, peritoneal dialysis; HD, hemodialysis; PY, person-year; CVE, cardiovascular event; AMI, acute myocardial infarction.

**Table 3:** Hazard of PD for clinical outcomes over hemodialysis according to the period.

	2004–2007 (n = 32,794)				2008–2011 (n = 30,518)				2012–2015 (n = 33,329)			
	Univariate		Multivariate		Univariate		Multivariate		Univariate		Multivariate	
	HR (95% CI)	*P*	HR (95% CI)	*P*	HR (95% CI)	*P*	HR (95% CI)	*P*	HR (95% CI)	*P*	HR (95% CI)	*P*
Mortality	1.18 (1.14–1.22)	<0.001	1.31 (1.27–1.36)	<0.001	0.84 (0.81–0.88)	<0.001	1.21 (1.16–1.27)	<0.001	0.63 (0.58–0.68)	<0.001	1.00 (0.91–1.09)	0.9
Nonfatal CVE	1.09 (1.03–1.16)	0.003	1.15 (1.09–1.23)	<0.001	0.87 (0.81–0.94)	<0.001	1.10 (1.02–1.19)	0.01	0.71 (0.63–0.8)	<0.001	0.92 (0.82–1.03)	0.15
AMI	1.20 (1.08–1.34)	0.001	1.28 (1.15–1.43)	<0.001	0.95 (0.82–1.11)	0.54	1.23 (1.06–1.43)	0.01	1.02 (0.84–1.25)	0.81	1.33 (1.09–1.63)	0.01
Any stroke	1.06 (0.99–1.13)	0.10	1.11 (1.04–1.19)	0.003	0.85 (0.78–0.93)	<0.001	1.07 (0.98–1.16)	0.13	0.62 (0.54–0.71)	<0.001	0.80 (0.70–0.92)	0.002
Ischemic	0.95 (0.83–1.10)	0.50	0.97 (0.85–1.12)	0.73	0.75 (0.63–0.91)	0.003	0.83 (0.69–1.00)	0.05	0.72 (0.55–0.95)	0.02	0.74 (0.56–0.98)	0.03
Hemorrhagic	1.06 (0.98–1.16)	0.15	1.13 (1.04–1.23)	0.003	0.87 (0.79–0.97)	0.01	1.14 (1.03–1.27)	0.01	0.57 (0.48–0.68)	<0.001	0.79 (0.66–0.95)	0.01

PD, peritoneal dialysis; HD, hemodialysis; HR, hazard ratio; CI confidence interval; CVE, cardiovascular event; AMI, acute myocardial infarction. In multivariate analysis, age, sex, type of insurance, income status, and CCI value were used as covariates.

**Figure 2:**
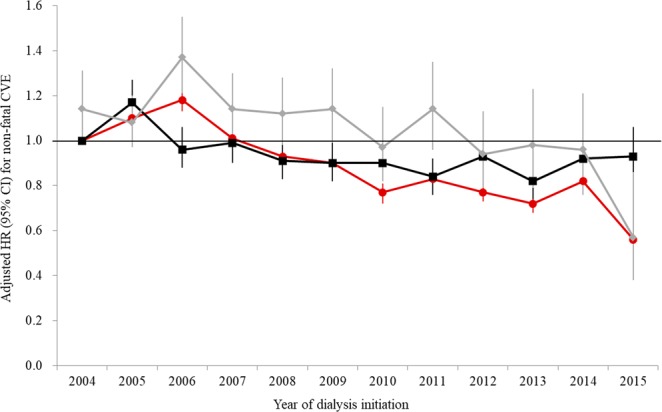
Secular trend of nonfatal CVE according to dialysis modality and the respective hazard of PD over HD. The black line indicates nonfatal CVE trends of HD, and the red line indicates that of PD according to the year of dialysis initiation. The gray line indicates the respective hazard ratio of PD over HD. Adjusted HRs and 95% CIs were calculated by multivariate Cox proportional hazard regression analysis, entering age, sex, insurance type, income status, and CCI value as covariates. HR, hazard ratio; CI, confidence interval; HD, hemodialysis; PD, peritoneal dialysis; CVE, cardiovascular event.

We compared the outcomes of nonfatal cardiovascular events (CVEs) according to dialysis modality. In the period of 2004 to 2007, the incidence rate of nonfatal CVE (37.3 per 1,000 person-years) in PD patients was higher than that in HD patients (33.1 per 1,000 person-years), as shown in Table 2, which was confirmed by the finding that HR (95% CI) of PD over HD for nonfatal CVE was 1.15 (1.09–1.23; *P* < 0.001) in multivariate Cox proportional hazard regression analysis (Table 3). Similar to mortality, however, the outcome of nonfatal CVE in PD patients seemed to be improved thereafter, as the hazard of nonfatal CVE of PD over HD was not statistically significant in the period of 2012 to 2015 (HR: 0.92, 95% CI: 0.82–1.03; *P* = 0.149). Unlike in HD patients, the outcome of nonfatal CVE in PD patients steadily improved as the study period progressed, and the hazard of nonfatal CVE of PD over HD presented as insignificant beginning in 2007, and was even significantly lower in year 2015 (Fig. 2). In the details of the components of nonfatal CVE (Table 3), PD was associated with an increased hazard for nonfatal acute myocardial infarction (AMI) as compared with HD throughout the study period. The hazard of PD for nonfatal ischemic stroke was lower than that of HD for the same, a finding that was statistically insignificant in the periods of 2004 to 2007 (P = 0.725) and 2008 to 2001 (P = 0.053), but which was significant in 2012 to 2015 (P = 0.033). Although PD was associated with an increased hazard of nonfatal hemorrhagic stroke in the former two periods, it was associated with a decreased hazard of nonfatal hemorrhagic stroke in the period of 2012 to 2015 as compared with HD, with a HR (95% CI) of 0.79 (0.66–0.95, *P* = 0.011).

We also analyzed characteristics in the crude incidence rates in the components of nonfatal CVE (Table 2). The crude incidence rate of nonfatal stroke was much higher than that of nonfatal AMI throughout the study period. Among cases of nonfatal stroke, the incidence rate of the hemorrhagic subtype was much higher than that of the ischemic subtype throughout the study period.

**Figure 3:**
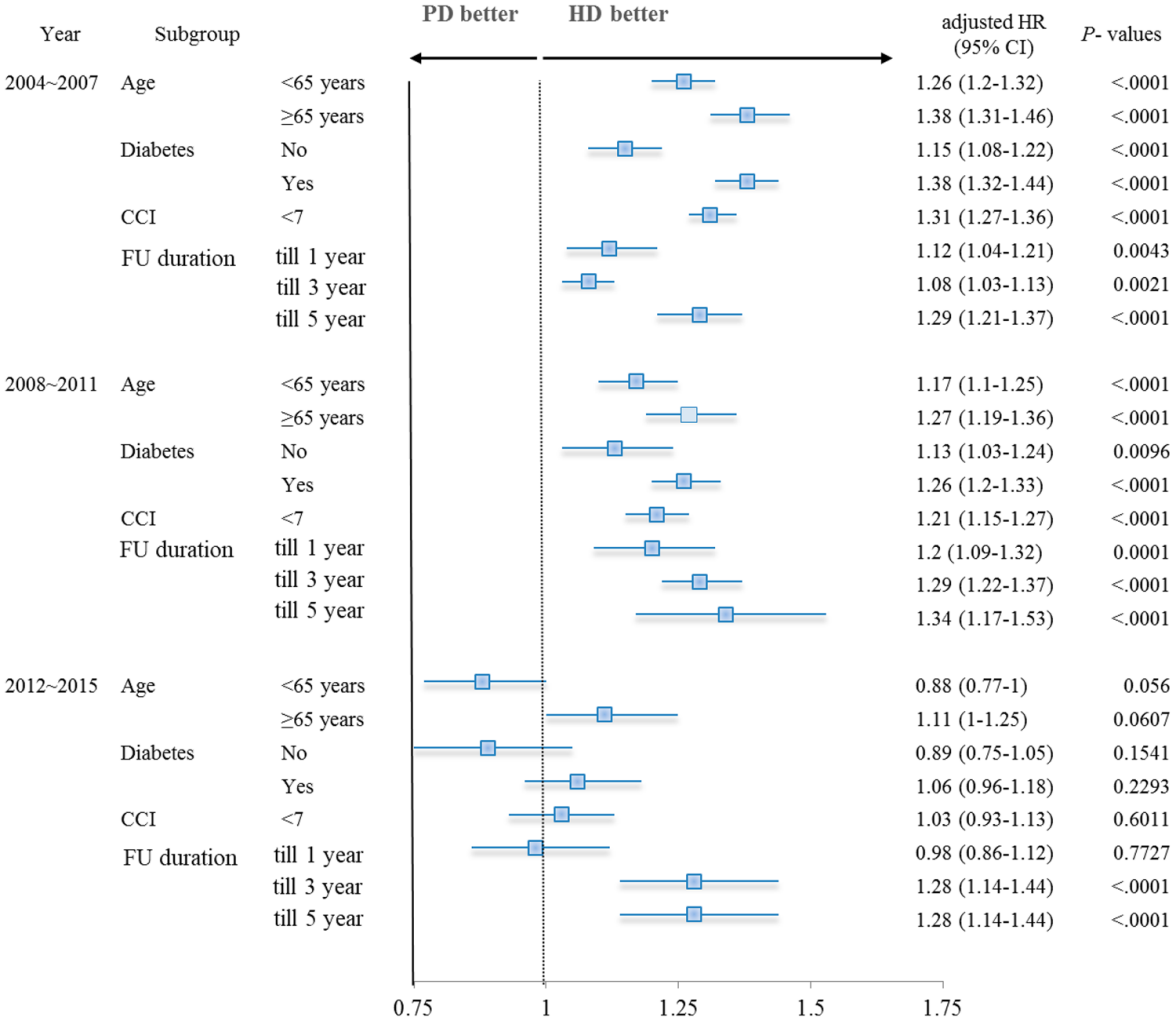
Subgroup analysis of the association between dialysis modality and patient mortality. Adjusted HRs and CIs were calculated by multivariate Cox proportional hazard regression analysis, entering age, sex, insurance type, income status, and CCI as covariates. HR, hazard ratio; CI, confidence interval; HD, hemodialysis; PD, peritoneal dialysis; Charlson Comorbidity Index, CCI; FU, follow up.

We performed subgroup analysis according to the following important effect modifiers: age, diabetes, comorbidity burden, and follow up duration (Fig. 3). In the period of 2004 to 2011, where the overall hazard of mortality of PD exceeded that of HD, the outcome of PD was consistently poorer than that of HD, regardless of the status of the effect modifiers. However, in the period of 2012 to 2015, the mortality of PD was comparable to that of HD, regardless of the status of age, diabetes, and comorbidity burden. Furthermore, although the hazard of PD over HD for mortality was insignificant when follow up was censored at one year, which became significant when follow up was censored at three or five year.

## Discussion

In Korea, the prevalence of HD has steeply increased, whereas that of PD has steadily decreased as time goes by^1^. Although the exact reason for the decline of PD in Korea has not been identified, the potential hazard of PD over HD for mortality may partly explain the recent decrease of PD prevalence in Korea^10^. However, PD is more economical than HD because it is not only cheaper^6^, but also more likely to be employed than HD^7^. PD has a beneficial effect in maintaining residual renal function^17^, which may more positively impact the survival rates of PD during the early dialysis period^18^ and after kidney transplantation^9^. According to recent registry data in Korea, the mortality of PD over HD seemed to be improved^16^, as has also been seen in other countries^11–14^. However, registry data cannot represent whole ESRD patients in Korea because not all dialysis centers nationwide participated in the registry. Therefore, a nationally representative study was warranted to confirm the possible improvement of PD mortality in Korea. With the current study, we aimed to identify secular trends of clinical outcomes in PD versus HD and to elucidate associated factors for the changes using a nationwide retrospective cohort of whole Korean ESRD patients who had begun dialysis during 2004 to 2015.

In the present study, we found that the PD patient survival became improved as the study period progressed. In the former two periods (years 2004–2011), PD was independently inferior to HD in terms of patient survival. However, the hazard of PD over HD for mortality has been nullified in the period of 2012 to 2015. In detail, the hazard of PD over HD for mortality was firstly insignificant in year 2013, and was significantly lower than HD in years 2014 to 2015. This improvement of patient survival with PD was in accordance with the results of the study by Mehrotra *et al*.^12^. Using the United States Renal Data System, they reported that the HR (95% CI) of PD over HD was 1.07 (1.04–1.11; *P* < 0.001) in the period of 1996 to 1998 and 1.08 (1.06–1.11; *P* < 0.001) in the period of 1999 to 2001, and was ultimately nullified in the period of 2002 to 2004 (HR: 1.03, 95% CI: 0.99–1.06, *P* = 0.10). Yeates *et al*.^13^ and Chang *et al*.^14^ also reported the occurrence of early inferior but later equivocal patient survival rates with PD over HD among Canadian (1991–2000 vs. 2001–2004) and Taiwanese (1997–2001 vs. 2002–2006) patients. The increased prescription of solutions with low-glucose degradation products and neutral pHs along with improved technique survival and peritonitis outcomes may have led to the improvement of PD patient survival in Korea^19,20^.

In the current study, the outcome of nonfatal CVE in PD was significantly poorer than that in HD in the former two periods (years 2004–2011). However, the hazard of nonfatal CVE with PD over HD became insignificant in 2012 to 2015, an occurrence that was largely attributed to the significantly lower hazard of nonfatal CVE with PD over HD in 2015. The trend of hazard with PD over HD for nonfatal CVE was mostly paralleled by that of nonfatal stroke, particularly the hemorrhagic subtype (Fig S1). In the general Korean population, the incidence of nonfatal stroke was higher than that of nonfatal AMI^21^, which was also true in the current study’s ESRD cohort. However, although the majority of nonfatal stroke cases involved the ischemic subtype in the general population^21^, the major subtype of nonfatal stroke in the present ESRD cohort was hemorrhagic, which was quite a unique feature to see in Korean ESRD patients^22^. Therefore, we postulated that the improved PD patient survival in this study may also be affected by the improved outcome of nonfatal hemorrhagic stroke, a major subtype of nonfatal stroke. The increased rates of atrial fibrillation and previous stroke in HD patients as well as the decreased use of antiplatelet agents and anticoagulants in PD patients may have impacted the nonhemorrhagic stroke rate.

Although the current study did not clearly demonstrate so, generally, young, nondiabetic patients with low comorbidity burdens are thought to be good candidates for PD^2^. Nevertheless, Korean ESRD patients with previous myocardial infarction, diabetes, and congestive heart failure tended to choose PD, which leads to an increased mortality risk^4^. In this study, more PD patients had diabetes and previous CAD than did HD patients in the period of 2004 to 2007. However, the rates of diabetes and previous CAD in HD patients increased thereafter. This improved risk profile led the reduced risk of nonfatal AMI development in PD patients throughout the study period. Since the increased risk of nonfatal AMI development throughout the study period was a major shortcoming of PD in this study, the good control of risk profiles and the resultant improvement of nonfatal AMI in PD patients may also affect the survival of these individuals.

This study has several limitations. First, the study was retrospective in nature, which limits the causal relationship. However, the study cohort was nationally representative and included all incident ESRD patients. Therefore, this limitation may be partially compensated for. Second, claims data can under- or overreport the true value. However, such misclassification may have an impact equally throughout the study period. Therefore, this weakness may ultimately have little effect on the study results. Third, the evaluation of a single nation with a single ethnicity may limit the generalizability of the present study to other parts of the world.

In conclusion, similar to other countries, PD patient survival in Korea has been significantly improved and the hazard of mortality of PD was significantly lower than that of HD during recent years. Improved outcomes of nonfatal CVE, particularly the hemorrhagic stroke subtype, may lead to further improvement.

## Methods

### Source of data

The National Health Insurance Service database (NHIS; Seoul, South Korea)—a public database related to health care utilization, health screening, sociodemographic variables, and mortality of the whole population of Korea—was used^23^. The NHIS database contains information pertaining to demographics and all the medical services rendered, along with the relevant diagnostic code(s) (as per the International Statistical Classification of Diseases and Related Health Problems, 10th edition (ICD-10) code) and all dispensed prescription medications.

### Study design

A nationwide retrospective cohort study based on the NHIS database was conducted. The source population included all of the patients with ESRD who underwent initial dialysis for at least two months between January 1, 2004 and December 31, 2015.

An eligible cohort of new patients treated with dialysis was identified among individuals who were dialyzed for two months or longer between January 1, 2004 and December 31, 2015. The date of the first dialysis was considered to be the initiation date. Individuals who had a claim for dialysis before the initiation date, who underwent renal transplantation before the initiation date or within three months after the initiation date, who died or were lost to follow-up within three months of the initiation date, and/or those who were not dialyzed within three months after the initiation date were excluded from the present study.

Eligible individuals were monitored until the diagnosis of cardiovascular disease, death, or kidney transplantation and followed until December 31, 2015. The protocol of the present study was approved by the ethics review committee of the National Evidence-based Healthcare Collaborating Agency (institutional review board number: NECA IRB 16–007–1). The study protocol adhered to the principles of the Declaration of Helsinki. Written informed consent was waived as the study did not infringe on patient privacy or health status.

### Case definition

Within the eligible cohort, we identified cases that claimed HD (V003, O7020, O9991) at three months after the initiation date of dialysis. The rationale for designating case definition at three months was to include patients who were finally treated with PD, even though they were usually treated initially by temporary HD. Therefore, patients who were treated with both HD and PD were identified as PD cases.

### Definitions and measurements

The comorbidities of interest were diabetes, hypertension, previous stroke, previous CAD, malignancy, liver and lung disease, atrial fibrillation, and hypothyroidism, which were chosen on the basis of suggestions by Charlson *et al*.^24^ to calculate the Charlson Comorbidity Index (CCI). These comorbidities were defined using ICD-10 codes according to the proposed algorithms of Quan *et al*.^25^ over a one-year period prior to initiation of dialysis therapy. Prescribed medications of interest were angiotensin II receptor blocker/angiotensin-converting enzyme inhibitors, other antihypertensive drugs, oral antidiabetic drugs, statin, antiplatelet agents, erythropoiesis-stimulating agents, and supplemental vitamin D. All medication users were defined as individuals who were prescribed medication at least once during the follow-up period after the index date of dialysis. Income status was classified into 20 classes according to the level of insurance premium; for reference, Level 1 denotes the bottom 5%, while level 20 denotes the top 5%.

The primary outcome of the current study was mortality, and secondary outcomes were nonfatal CVEs. Mortality was ascertained using data of the Korea National Statistical Office for the cause of death, after excluding deaths due to aging, Parkinson’s disease, suicide, and accidents. Nonfatal CVEs included nonfatal AMI and stroke, which were defined as the first hospitalization with I21-I22 code or I60-I68 code, respectively, after dialysis initiation that did not end in death.

### Statistical analysis

Continuous variables are expressed as the mean ± standard deviation and categorical variables are expressed as the percentage. Differences were analyzed using the Student’s t-test for continuous variables and the chi-squared test for categorical variables. HRs and 95% CIs of PD over HD for mortality and nonfatal CVEs were calculated using a Cox proportional hazards regression model. The assumption of proportional hazard was tested by log-minus-log plot for categorical variables and interaction analysis with time covariate using time-dependent Cox regression for continuous variables. In multivariate analysis, age, sex, type of insurance, income status, and CCI were used as covariates. Death was treated as censoring when we calculated the hazard of nonfatal CVEs. A *P*-value of less than 0.05 was considered to be statistically significant, and all statistical analyses were performed using SAS version 9.3 (SAS Institute, Cary, NC, USA).

## Supplementary information

SUPPLEMENTARY MATERIALS

## Data Availability

Available as supplementary material when accepted

## References

[1] Jin DC (2015). Lessons from 30 years’ data of Korean end-stage renal disease registry, 1985–2015. Kidney Res Clin Pract.

[2] Khawar O, Kalantar-Zadeh K, Lo WK, Johnson D, Mehrotra R (2007). Is the declining use of long-term peritoneal dialysis justified by outcome data?. Clin J Am Soc Nephrol.

[3] Jin DC (2015). Major changes and improvements of dialysis therapy in Korea: review of end-stage renal disease registry. Korean J Intern Med.

[4] Kim HJ (2017). The pattern of choosing dialysis modality and related mortality outcomes in Korea: a national population-based study. Korean J Intern Med.

[5] Ghaffari A (2013). PD First: peritoneal dialysis as the default transition to dialysis therapy. Semin Dial.

[6] Kim SH, Jo MW, Go DS, Ryu DR, Park J (2017). Economic burden of chronic kidney disease in Korea using national sample cohort. J Nephrol.

[7] Muehrer RJ (2011). Factors affecting employment at initiation of dialysis. Clin J Am Soc Nephrol.

[8] Iyasere OU (2016). Quality of Life and Physical Function in Older Patients on Dialysis: A Comparison of Assisted Peritoneal Dialysis with Hemodialysis. Clin J Am Soc Nephrol.

[9] Song SH (2016). Outcomes of Kidney Recipients According to Mode of Pretransplantation Renal Replacement Therapy. Transplant Proc.

[10] Kim H (2014). A population-based approach indicates an overall higher patient mortality with peritoneal dialysis compared to hemodialysis in Korea. Kidney Int.

[11] Vonesh EF, Moran J (1999). Mortality in end-stage renal disease: a reassessment of differences between patients treated with hemodialysis and peritoneal dialysis. J Am Soc Nephrol.

[12] Mehrotra R, Chiu YW, Kalantar-Zadeh K, Bargman J, Vonesh E (2011). Similar outcomes with hemodialysis and peritoneal dialysis in patients with end-stage renal disease. Arch Intern Med.

[13] Yeates K (2012). Hemodialysis and peritoneal dialysis are associated with similar outcomes for end-stage renal disease treatment in Canada. Nephrol Dial Transplant.

[14] Chang YK (2012). A comparative assessment of survival between propensity score-matched patients with peritoneal dialysis and hemodialysis in Taiwan. Medicine (Baltimore).

[15] Ryu JH (2015). Improving survival rate of Korean patients initiating dialysis. Yonsei Med J.

[16] Choi JY (2013). Survival advantage of peritoneal dialysis relative to hemodialysis in the early period of incident dialysis patients: a nationwide prospective propensity-matched study in Korea. PloS one.

[17] Misra M (2001). Effect of cause and time of dropout on the residual GFR: a comparative analysis of the decline of GFR on dialysis. Kidney Int.

[18] Kim H, Ryu DR (2017). A prime determinant in selecting dialysis modality: peritoneal dialysis patient survival. Kidney Res Clin Pract.

[19] Lee HY (2006). Changing prescribing practice in CAPD patients in Korea: increased utilization of low GDP solutions improves patient outcome. Nephrol Dial Transplant.

[20] Han SH (2007). Improving outcome of CAPD: twenty-five years’ experience in a single Korean center. Perit Dial Int.

[21] Jung CH (2017). Improved trends in cardiovascular complications among subjects with type 2 diabetes in Korea: a nationwide study (2006–2013). Cardiovasc Diabetol.

[22] Kuo CC (2012). Haemodialysis and the risk of stroke: A population-based cohort study in Taiwan, a country of high incidence of end-stage renal disease. Nephrology (Carlton).

[23] Cheol Seong S (2017). Data Resource Profile: The National Health Information Database of the National Health Insurance Service in South Korea. Int J Epidemiol.

[24] Charlson ME, Pompei P, Ales KL, MacKenzie CR (1987). A new method of classifying prognostic comorbidity in longitudinal studies: development and validation. J Chronic Dis.

[25] Quan H (2005). Coding algorithms for defining comorbidities in ICD-9-CM and ICD-10 administrative data. Med Care.

