# Supplementary material for: Comparative study of peritoneal dialysis versus hemodialysis on the clinical outcomes in Korea: a population-based approach
Source: Sci Rep. 2019 Apr 11;9:5905. doi: 10.1038/s41598-019-42508-z (PMC6459886; doi:10.1038/s41598-019-42508-z)
Supplement: Supplementary file 1 — SUPPLEMENTARY MATERIALS [file 41598_2019_42508_MOESM1_ESM.pdf]

Comparative study of peritoneal dialysis versus hemodialysis on the clinical outcomes in Korea: a population-based approach

Sung Woo Lee, MD, PhD[1]†, Na Rae Lee, MPH[2]†, Soo Kyung Son, MHS[2], Jimin Kim, MPH[2], Ah Ram Sul, PhD[2], Yunjung Kim, MPH[2]*, Jung Tak Park, MD, PhD[3], Jung Pyo Lee, MD, PhD[4,5], Dong-Ryeol Ryu, MD, PhD[6]*

[1]*Department of Nephrology, Internal Medicine, Nowon Eulji Medical center, Eulji University, Seoul, Korea*

[2]*National Evidence-based Healthcare Collaborating Agency*

[3]*Department of Internal Medicine, College of Medicine, Yonsei University, Seoul, Republic of Korea*

[4] *Department of Internal Medicine, Seoul National University Boramae Medcal Center, Seoul, Korea*

[5]*Department of Internal Medicine, Seoul National University College of Medicine, Seoul, Korea*

[6]*Department of Internal Medicine, School of Medicine, Ewha Womans University, Seoul, Korea*

†These authors contributed equally to this work and should be considered co-first authors.

*These authors contributed equally to this work and should be considered co-corresponding authors.

Supplemental Tables

Table S1. Secular trend of mortality according to dialysis modality and the respective hazard of PD over HD

Year	Respective HR of PD: HD	PD	HD
	Adjusted HR (95% CI)	Adjusted HR (95% CI)	Adjusted HR (95% CI)
2004	1.41 (1.31–1.52)	1.00	1.00
2005	1.33 (1.25–1.41)	1.05 (0.97–1.14)	1.13 (1.08–1.19)
2006	1.32 (1.23–1.42)	0.86 (0.79–0.94)	0.93 (0.88–0.98)
2007	1.29 (1.19–1.39)	0.83 (0.76–0.91)	0.92 (0.87–0.97)
2008	1.30 (1.20–1.41)	0.74 (0.68–0.82)	0.83 (0.79–0.88)
2009	1.22 (1.12–1.33)	0.68 (0.61–0.75)	0.81 (0.77–0.86)
2010	1.10 (0.99–1.22)	0.59 (0.53–0.66)	0.80 (0.75–0.84)
2011	1.15 (1.03–1.29)	0.60 (0.53–0.68)	0.76 (0.72–0.81)
2012	1.14 (1.01–1.29)	0.57 (0.50–0.65)	0.73 (0.69–0.78)
2013	1.04 (0.89–1.22)	0.49 (0.42–0.58)	0.67 (0.63–0.71)
2014	0.71 (0.56–0.89)	0.33 (0.26–0.41)	0.61 (0.56–0.65)
2015	0.59 (0.39–0.92)	0.36 (0.23–0.55)	0.74 (0.67–0.83)

PD, peritoneal dialysis; HD, hemodialysis; HR, hazard ratio; CI, confidence interval. Adjusted HRs and 95% CIs were calculated by multivariate Cox proportional hazard regression analysis, entering age, sex, insurance type, income status, and CCI value as covariates.

Table S2. Secular trend of nonfatal CVE according to dialysis modality and the respective hazard of PD over HD

Year	Respective HR of PD: HD	PD	HD
	Adjusted HR (95% CI)	Adjusted HR (95% CI)	Adjusted HR (95% CI)
2004	1.14 (1.00–1.31)	1.00	1.00
2005	1.08 (0.97–1.20)	1.10 (0.95–1.28)	1.17 (1.07–1.27)
2006	1.37 (1.21–1.55)	1.18 (1.01–1.38)	0.96 (0.88–1.06)
2007	1.14 (0.99–1.30)	1.01 (0.85–1.19)	0.99 (0.90–1.09)
2008	1.12 (0.98–1.28)	0.93 (0.79–1.10)	0.91 (0.83–1.00)
2009	1.14 (0.99–1.32)	0.90 (0.75–1.07)	0.90 (0.82–0.99)
2010	0.97 (0.82–1.15)	0.77 (0.64–0.93)	0.90 (0.82–0.99)
2011	1.14 (0.96–1.35)	0.83 (0.68–1.01)	0.84 (0.76–0.92)
2012	0.94 (0.79–1.13)	0.77 (0.63–0.95)	0.93 (0.85–1.03)
2013	0.98 (0.79–1.23)	0.72 (0.56–0.91)	0.82 (0.74–0.91)
2014	0.96 (0.76–1.21)	0.82 (0.64–1.05)	0.92 (0.82–1.02)
2015	0.57 (0.38–0.86)	0.56 (0.37–0.85)	0.93 (0.81–1.06)

CVE, cardiovascular event; PD, peritoneal dialysis; HD, hemodialysis; HR, hazard ratio; CI, confidence interval. Adjusted HRs and 95% CIs were calculated by multivariate Cox proportional hazard regression analysis, entering age, sex, insurance type, income status, and CCI value as covariates.

Table S3. Secular trend of nonfatal AMI according to dialysis modality and the respective hazard of PD over HD

Year	Respective HR of PD: HD	PD	HD
	Adjusted HR (95% CI)	Adjusted HR (95% CI)	Adjusted HR (95% CI)
2004	1.33 (1.04–1.69)	1.00	1.00
2005	1.33 (1.10–1.61)	1.20 (0.92–1.57)	1.17 (1.00–1.38)
2006	1.55 (1.23–1.95)	1.10 (0.83–1.45)	0.94 (0.78–1.12)
2007	1.05 (0.81–1.37)	0.81 (0.59–1.11)	1.02 (0.85–1.22)
2008	1.34 (1.02–1.76)	0.78 (0.57–1.07)	0.77 (0.64–0.93)
2009	1.16 (0.86–1.56)	0.67 (0.48–0.94)	0.81 (0.67–0.98)
2010	1.20 (0.87–1.65)	0.69 (0.48–0.99)	0.80 (0.66–0.96)
2011	1.23 (0.88–1.70)	0.70 (0.49–1.01)	0.77 (0.64–0.93)
2012	1.34 (0.98–1.83)	0.86 (0.60–1.23)	0.89 (0.74–1.08)
2013	1.39 (0.94–2.06)	0.80 (0.53–1.21)	0.72 (0.59–0.89)
2014	1.53 (1.03–2.27)	0.95 (0.62–1.45)	0.84 (0.68–1.03)
2015	0.71 (0.32–1.56)	0.47 (0.22–1.02)	0.82 (0.63–1.07)

AMI, acute myocardial infarction; PD, peritoneal dialysis; HD, hemodialysis; HR, hazard ratio; CI, confidence interval. Adjusted HRs and 95% CIs were calculated by multivariate Cox proportional hazard regression analysis, entering age, sex, insurance type, income status, and CCI value as covariates.

Table S4. Secular trend of nonfatal stroke according to dialysis modality and the respective hazard of PD over HD

Year	Respective HR of PD: HD	PD	HD
	Adjusted HR (95% CI)	Adjusted HR (95% CI)	Adjusted HR (95% CI)
2004	1.09 (0.93–1.27)	1.00	1.00
2005	1.00 (0.89–1.13)	1.05 (0.89–1.25)	1.16 (1.06–1.28)
2006	1.30 (1.13–1.49)	1.18 (0.99–1.41)	0.96 (0.86–1.07)
2007	1.18 (1.02–1.37)	1.08 (0.90–1.30)	0.97 (0.87–1.07)
2008	1.06 (0.91–1.23)	0.96 (0.79–1.15)	0.95 (0.85–1.05)
2009	1.13 (0.96–1.33)	0.95 (0.78–1.16)	0.92 (0.83–1.02)
2010	0.93 (0.77–1.12)	0.80 (0.64–1.00)	0.93 (0.83–1.03)
2011	1.13 (0.94–1.37)	0.89 (0.71–1.10)	0.87 (0.78–0.97)
2012	0.81 (0.65–1.00)	0.71 (0.55–0.91)	0.94 (0.84–1.05)
2013	0.88 (0.68–1.14)	0.68 (0.51–0.91)	0.85 (0.76–0.95)
2014	0.82 (0.62–1.08)	0.76 (0.56–1.02)	0.93 (0.82–1.04)
2015	0.55 (0.35–0.89)	0.61 (0.38–0.98)	0.95 (0.82–1.10)

PD, peritoneal dialysis; HD, hemodialysis; HR, hazard ratio; CI, confidence interval. Adjusted HRs and 95% CIs were calculated by multivariate Cox proportional hazard regression analysis, entering age, sex, insurance type, income status, and CCI value as covariates.

Table S5. Secular trend of nonfatal ischemic stroke according to dialysis modality and the respective hazard of PD over HD

Year	Respective HR of PD: HD	PD	HD
	Adjusted HR (95% CI)	Adjusted HR (95% CI)	Adjusted HR (95% CI)
2004	1.11 (0.93–1.33)	1.00	1.00
2005	1.02 (0.88–1.19)	1.02 (0.83–1.25)	1.14 (1.02–1.28)
2006	1.40 (1.19–1.65)	1.16 (0.94–1.43)	0.90 (0.79–1.02)
2007	1.13 (0.93–1.36)	0.92 (0.73–1.16)	0.89 (0.79–1.01)
2008	1.12 (0.93–1.35)	0.86 (0.69–1.09)	0.83 (0.73–0.94)
2009	1.15 (0.95–1.41)	0.87 (0.69–1.11)	0.83 (0.73–0.95)
2010	1.03 (0.82–1.3)	0.73 (0.56–0.95)	0.76 (0.67–0.87)
2011	1.22 (0.97–1.54)	0.80 (0.61–1.04)	0.72 (0.63–0.82)
2012	0.81 (0.61–1.07)	0.58 (0.42–0.79)	0.77 (0.67–0.88)
2013	0.84 (0.60–1.17)	0.56 (0.39–0.81)	0.74 (0.65–0.85)
2014	0.83 (0.57–1.20)	0.57 (0.38–0.84)	0.71 (0.61–0.83)
2015	0.55 (0.29–1.01)	0.49 (0.26–0.90)	0.81 (0.67–0.97)

PD, peritoneal dialysis; HD, hemodialysis; HR, hazard ratio; CI, confidence interval. Adjusted HRs and 95% CIs were calculated by multivariate Cox proportional hazard regression analysis, entering age, sex, insurance type, income status, and CCI value as covariates.

Table S6. Secular trend of nonfatal hemorrhagic stroke according to dialysis modality, and the respective hazard of PD over HD

Year	Respective HR of PD: HD	PD	HD
	Adjusted HR (95% CI)	Adjusted HR (95% CI)	Adjusted HR (95% CI)
2004	0.84 (0.60–1.17)	1.00	1.00
2005	0.88 (0.69–1.13)	1.17 (0.81–1.70)	1.14 (0.95–1.37)
2006	1.18 (0.89–1.58)	1.33 (0.91–1.95)	0.93 (0.75–1.16)
2007	1.10 (0.82–1.49)	1.40 (0.94–2.08)	1.02 (0.83–1.27)
2008	0.93 (0.68–1.27)	1.20 (0.80–1.80)	1.06 (0.86–1.32)
2009	0.89 (0.60–1.31)	0.88 (0.55–1.40)	0.86 (0.68–1.07)
2010	0.60 (0.39–0.91)	0.85 (0.52–1.40)	1.15 (0.93–1.43)
2011	0.90 (0.59–1.37)	0.99 (0.61–1.62)	0.94 (0.75–1.18)
2012	0.66 (0.42–1.05)	0.92 (0.54–1.56)	1.07 (0.85–1.34)
2013	1.07 (0.63–1.80)	1.02 (0.58–1.80)	0.81 (0.63–1.04)
2014	0.69 (0.40–1.18)	1.14 (0.63–2.08)	1.30 (1.03–1.66)
2015	0.52 (0.21–1.33)	0.90 (0.35–2.29)	1.06 (0.77–1.45)

PD, peritoneal dialysis; HD, hemodialysis; HR, hazard ratio; CI, confidence interval. Adjusted HRs and 95% CIs were calculated by multivariate Cox proportional hazard regression analysis, entering age, sex, insurance type, income status, and CCI value as covariates.

Table S7. Baseline characteristics of the study population according to the study period after propensity score matching

	2004–2007 (n = 15,090)			2008–2011 (n = 11,936)			2012–2015 (n =9,398)		
	PD (n = 7,545)	HD (n = 7,545)	*P*	PD (n = 5,968)	HD (n = 5,968)	*P*	PD (n = 4,699)	HD (n = 4,699)	*P*
Age (years)	53.9±13.6	54±13.7	0.84	54.2±14	54.4±13.9	0.45	54.6±14.1	54.7±14	0.66
Male sex (n, %)	4,195 (55.6)	4,200 (55.7)	0.93	3,496 (58.6)	3,499 (58.6)	0.96	2,763 (58.8)	2,782 (59.2)	0.69
Medical aid (n, %)	1,424 (18.9)	1,377 (18.3)	0.33	583 (9.8)	559 (9.4)	0.46	321 (6.8)	279 (5.9)	0.08
Income status	8.5±7.1	8.3±7.1	0.16	10.3±6.6	10.1±6.6	0.04	10.4±6.5	10.3±6.3	0.17
Diabetes (n, %)	4,088 (54.2)	4,088 (54.2)	1.00	3,486 (58.4)	3,486 (58.4)	1.00	2,791 (59.4)	2,791 (59.4)	1.00
Hypertension (n, %)	7,338 (97.3)	7,338 (97.3)	1.00	5,915 (99.1)	5,915 (99.1)	1.00	4,637 (98.7)	4,637 (98.7)	1.00
Previous stroke (n, %)	786 (10.4)	817 (10.8)	0.41	701 (11.8)	717 (12)	0.65	498 (10.6)	555 (11.8)	0.06
Previous CAD (n, %)	786 (10.4)	590 (7.8)	<.001	585 (9.8)	531 (8.9)	0.09	426 (9.1)	374 (8)	0.05
Malignancy (n, %)	194 (2.6)	180 (2.4)	0.46	195 (3.3)	153 (2.6)	0.02	198 (4.2)	170 (3.6)	0.14
Liver disease (n, %)	813 (10.8)	891 (11.8)	0.04	744 (12.5)	780 (13.1)	0.32	559 (11.9)	620 (13.2)	0.06
Lung disease (n, %)	665 (8.8)	655 (8.7)	0.77	697 (11.7)	656 (11)	0.24	562 (12)	577 (12.3)	0.64

Atrial fibrillation (n, %)	102 (1.4)	70 (0.9)	0.01	87 (1.5)	77 (1.3)	0.43	78 (1.7)	81 (1.7)	0.81
Hypothyroidism (n, %)	168 (2.2)	151 (2)	0.34	181 (3)	214 (3.6)	0.09	196 (4.2)	182 (3.9)	0.46
CCI score	3.9±1.7	3.8±1.7	0.80	4.2±1.7	4.2±1.7	0.58	4.1±1.7	4.1±1.7	0.85
1–3	3,502 (46.4)	3,507 (46.5)	0.25	2,366 (39.6)	2,397 (40.2)	0.22	1,929 (41.1)	1,933 (41.1)	0.89
4–6	3,543 (47)	3,587 (47.5)	0.25	3,066 (51.4)	3,088 (51.7)	0.22	2,358 (50.2)	2,367 (50.4)	0.89
≥ 7	500 (6.6)	451 (6)	0.25	536 (9)	483 (8.1)	0.22	412 (8.8)	399 (8.5)	0.89
ACEI/ARB (n, %)	7,179 (95.2)	7,069 (93.7)	<.001	5,649 (94.7)	5,575 (93.4)	0.00	4,173 (88.8)	4,096 (87.2)	0.01
Other anti-HTN drugs (n, %)	7,493 (99.3)	7,436 (98.6)	<.001	5,933 (99.4)	5,901 (98.9)	0.00	4,610 (98.1)	4,571 (97.3)	0.01
OAD (n, %)	3,181 (42.2)	3,245 (43)	0.29	2,470 (41.4)	2,433 (40.8)	0.49	2,007 (42.7)	1,910 (40.7)	0.04
Statin (n, %)	5,254 (69.6)	4,415 (58.5)	<.001	4,367 (73.2)	3,630 (60.8)	<.001	3,089 (65.7)	2,638 (56.1)	<.001
Anti-platelet agents (n, %)	5,889 (78.1)	6,750 (89.5)	<.001	4,316 (72.3)	5,135 (86)	<.001	2,455 (52.3)	3,358 (71.5)	<.001
Anti-coagulants (n, %)	614 (8.1)	755 (10)	<.001	400 (6.7)	502 (8.4)	0.00	193 (4.1)	273 (5.8)	<.001

PD, peritoneal dialysis; HD, hemodialysis; CAD, coronary artery disease; ACEI/ARB, angiotensinogen-converting enzyme inhibitor/angiotensin receptor blocker; HTN, hypertension; OAD, oral antidiabetic drug; ESA, erythropoiesis-stimulating agent; CCI, Charlson Comorbidity Index. Values are expressed as the mean ± standard deviation for continuous variables and n (%) for categorical variables. Differences were evaluated by t-tests for continuous variables and chi-squared tests for categorical variables.

Strata Propensity-score matching was used to assemble patient cohort with similar baseline characteristics.

Stratified factors: diabetes mellitus, hypertension

Matching factors: age, sex, medical aid, income status, previous stroke, previous CAD, malignancy, liver disease, lung disease, atrial fibrillation, hypothyroidism

Table S8. Crude incidence rates of major clinical outcomes of dialysis modality according to the study period after propensity score matching

	2004–2007 (n = 15,090)				2008–2011 (n = 11,936)				2012–2015 (n =9,398)			
	PD (n = 7,545)		HD (n = 7,545)		PD (n = 5,968)		HD (n = 5,968)		PD (n = 4,699)		HD (n = 4,699)	
	N (%)	1,000 PY	N (%)	1 000 PY	N (%)	1,000 PY	N (%)	1,000 PY	N (%)	1,000 PY	N (%)	1,000 PY
Mortality	4,319 (57.2)	105.7	3,953 (52.4)	83.6	2,215 (37.1)	87.6	1,923 (32.2)	70	582 (12.4)	66	553 (11.8)	62.5
Nonfatal CVE	1,379 (18.3)	37.3	1,408 (18.7)	32.9	842 (14.1)	35.8	849 (14.2)	33.4	334 (7.1)	39.6	374 (8)	44.7
AMI	414 (5.5)	10.4	377 (5)	8.2	219 (3.7)	8.8	207 (3.5)	7.7	116 (2.5)	13.4	84 (1.8)	9.6
Any stroke	1,048 (13.9)	27.7	1,094 (14.5)	25	657 (11)	27.5	666 (11.2)	25.8	230 (4.9)	26.9	299 (6.4)	35.3
Ischemic stroke	717 (9.5)	18.5	747 (9.9)	16.6	440 (7.4)	18.1	431 (7.2)	16.3	136 (2.9)	15.7	164 (3.5)	19
Hemorrhagic stroke	242 (3.2)	6.0	278 (3.7)	6	134 (2.3)	5.4	171 (2.9)	6.3	58 (1.2)	6.6	80 (1.7)	9.1

PD, peritoneal dialysis; HD, hemodialysis; PY, person-year; CVE, cardiovascular event; AMI, acute myocardial infarction.

Table S9. Hazard of PD for clinical outcomes over hemodialysis according to the period after propensity score matching

	2004–2007 (n = 15,090)				2008–2011 (n = 11,936)				2012–2015 (n =9,398)			
	Univariate		Multivariate		Univariate		Multivariate		Univariate		Multivariate	
	HR (95% CI)	*P*	HR (95% CI)	*P*	HR (95% CI)	*P*	HR (95% CI)	*P*	HR (95% CI)	*P*	HR (95% CI)	*P*
Mortality	1.26 (1.21-1.31)	<.001	1.26 (1.21-1.32)	<.001	1.25 (1.18-1.33)	<.001	1.29 (1.22-1.38)	<.001	1.06 (0.94-1.19)	0.35	1.07 (0.95-1.2)	0.28
Nonfatal CVE	1.1 (1.02-1.18)	0.01	1.1 (1.02-1.19)	0.01	1.05 (0.96-1.16)	0.28	1.07 (0.97-1.18)	0.16	0.89 (0.77-1.03)	0.11	0.89 (0.77-1.03)	0.12
AMI	1.23 (1.07-1.41)	0.00	1.23 (1.07-1.42)	0.00	1.14 (0.94-1.38)	0.19	1.16 (0.96-1.41)	0.12	1.39 (1.05-1.84)	0.02	1.4 (1.05-1.85)	0.02
Any stroke	1.08 (0.99-1.17)	0.08	1.08 (0.99-1.18)	0.07	1.05 (0.94-1.17)	0.39	1.06 (0.96-1.19)	0.26	0.76 (0.64-0.91)	0.00	0.76 (0.64-0.91)	0.00
Ischemic	1.08 (0.97-1.19)	0.15	1.08 (0.98-1.2)	0.14	1.09 (0.95-1.24)	0.23	1.11 (0.97-1.26)	0.13	0.83 (0.66-1.04)	0.10	0.83 (0.66-1.04)	0.10
Hemorrhagic	0.98 (0.83-1.17)	0.85	0.98 (0.83-1.17)	0.86	0.84 (0.67-1.05)	0.13	0.85 (0.68-1.07)	0.16	0.73 (0.52-1.02)	0.06	0.73 (0.52-1.02)	0.06

PD, peritoneal dialysis; HD, hemodialysis; HR, hazard ratio; CI confidence interval; CVE, cardiovascular event; AMI, acute myocardial infarction. In multivariate analysis, age, sex, type of insurance, income status, and CCI value were used as covariates.

Table S10. Hazard of PD for clinical outcomes over hemodialysis according to the period using competing risk analysis

	2004–2007 (n = 32,794)				2008–2011 (n = 30,518)				2012–2015 (n = 33,329)			
	Univariate		Multivariate		Univariate		Multivariate		Univariate		Multivariate	
	HR (95% CI)	*P*	HR (95% CI)	*P*	HR (95% CI)	*P*	HR (95% CI)	*P*	HR (95% CI)	*P*	HR (95% CI)	*P*
Mortality	1.1 (1.07-1.14)	<.001	1.36 (1.31-1.41)	<.001	0.8 (0.77-0.84)	<.001	1.27 (1.21-1.33)	<.001	0.62 (0.57-0.67)	<.001	1.05 (0.96-1.15)	0.26
Nonfatal CVE	1.09 (1.03–1.16)	0.003	1.15 (1.09–1.23)	<0.001	0.87 (0.81–0.94)	<0.001	1.10 (1.02–1.19)	0.01	0.71 (0.63–0.8)	<0.001	0.92 (0.82–1.03)	0.15
AMI	1.20 (1.08–1.34)	0.001	1.28 (1.15–1.43)	<0.001	0.95 (0.82–1.11)	0.54	1.23 (1.06–1.43)	0.01	1.02 (0.84–1.25)	0.81	1.33 (1.09–1.63)	0.01
Any stroke	1.06 (0.99–1.13)	0.10	1.11 (1.04–1.19)	0.003	0.85 (0.78–0.93)	<0.001	1.07 (0.98–1.16)	0.13	0.62 (0.54–0.71)	<0.001	0.80 (0.70–0.92)	0.002
Ischemic	0.95 (0.83–1.10)	0.50	0.97 (0.85–1.12)	0.73	0.75 (0.63–0.91)	0.003	0.83 (0.69–1.00)	0.05	0.72 (0.55–0.95)	0.02	0.74 (0.56–0.98)	0.03
Hemorrhagic	1.06 (0.98–1.16)	0.15	1.13 (1.04–1.23)	0.003	0.87 (0.79–0.97)	0.01	1.14 (1.03–1.27)	0.01	0.57 (0.48–0.68)	<0.001	0.79 (0.66–0.95)	0.01

PD, peritoneal dialysis; HD, hemodialysis; HR, hazard ratio; CI confidence interval; CVE, cardiovascular event; AMI, acute myocardial infarction. In multivariate analysis, age, sex, type of insurance, income status, and CCI value were used as covariates.

Table S11. Hazard of PD for clinical outcomes over hemodialysis according to the period after adding more covariates

	2004–2007 (n = 32,794)				2008–2011 (n = 30,518)				2012–2015 (n = 33,329)			
	Multivariate 1		Multivariate 2		Multivariate 1		Multivariate 2		Multivariate 1		Multivariate 2	
	HR (95% CI)	*P*	HR (95% CI)	*P*	HR (95% CI)	*P*	HR (95% CI)	*P*	HR (95% CI)	*P*	HR (95% CI)	*P*
Mortality	1.39 (1.34-1.44)	<.0001	1.37 (1.32-1.42)	<.0001	1.29 (1.23-1.35)	<.0001	1.25 (1.19-1.31)	<.0001	1.05 (0.97-1.15)	0.23	1.03 (0.95-1.13)	0.48
Nonfatal CVE	1.17 (1.1-1.25)	<.0001	1.15 (1.08-1.23)	<.0001	1.11 (1.03-1.19)	0.01	1.09 (1.01-1.17)	0.03	0.96 (0.86-1.08)	0.51	0.93 (0.82-1.04)	0.19
AMI	1.22 (1.09-1.37)	0.00	1.23 (1.1-1.38)	0.00	1.14 (0.98-1.33)	0.08	1.15 (0.99-1.34)	0.07	1.34 (1.1-1.64)	0.00	1.26 (1.03-1.54)	0.02
Any stroke	1.16 (1.08-1.25)	<.0001	1.13 (1.05-1.21)	0.00	1.1 (1.01-1.2)	0.03	1.07 (0.98-1.17)	0.11	0.86 (0.74-0.98)	0.03	0.83 (0.72-0.95)	0.01
Ischemic	1.22 (1.12-1.33)	<.0001	1.16 (1.06-1.26)	0.00	1.18 (1.06-1.31)	0.00	1.15 (1.03-1.28)	0.01	0.89 (0.74-1.06)	0.18	0.82 (0.68-0.97)	0.03
Hemorrhagic	0.96 (0.83-1.11)	0.57	0.98 (0.85-1.14)	0.84	0.83 (0.68-1)	0.05	0.85 (0.7-1.02)	0.08	0.68 (0.51-0.9)	0.01	0.77 (0.58-1.02)	0.07

PD, peritoneal dialysis; HD, hemodialysis; HR, hazard ratio; CI confidence interval; CVE, cardiovascular event; AMI, acute myocardial infarction.

M1: age, sex, type of insurance, income status, and CCI value, Diabetes Hypertension, Previous stroke, Previous CAD, Malignancy, Liver disease, Lung disease, Atrial fibrillation, Hypothyroidism, ACEI/ARB, Other anti-HTN drugs, OAD, Statin, Anti-platelet agents, Anti-coagulants

M2: age, sex, type of insurance, income status, and CCI value, Diabetes Hypertension, Previous stroke, Previous CAD, Malignancy, Liver disease, Lung disease, Atrial fibrillation, Hypothyroidism

Figure S1. Secular trend of components of nonfatal CVE according to dialysis modality and the respective hazard of PD over HD. Outcomes were AMI in panel A, any stroke in panel B, hemorrhagic stroke in panel C, and ischemic stroke in panel D. The black line indicates trends of the respective outcomes of HD, while the red line indicates those of PD according to the year of dialysis initiation. The gray line indicates the respective hazard ratio of PD over HD. Adjusted HRs and 95% CIs were calculated by multivariate Cox proportional hazard regression analysis, entering age, sex, insurance type, income status, and CCI value as covariates. HR, hazard ratio; CI, confidence interval; HD, hemodialysis; PD, peritoneal dialysis; CVE, cardiovascular event; AMI, acute myocardial infarction.

Figure S1-A

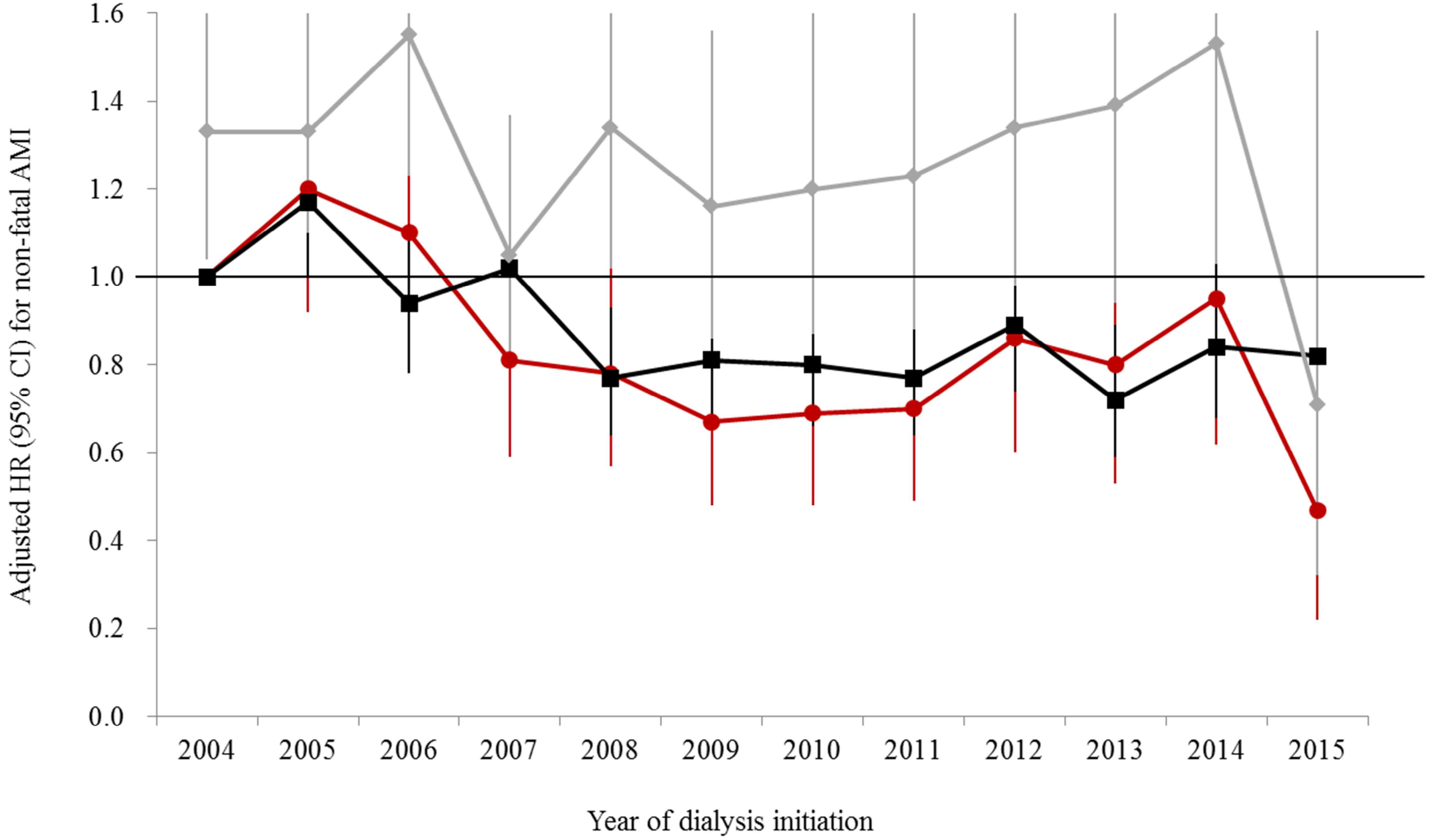

1.6
1.4
1.2
1.0
0.8
0.6
0.4
0.2
0.0
Adjusted HR (95% CI) for non-fatal AMI
2004
2005
2006
2007
2008
2009
2010
2011
2012
2013
2014
2015
Year of dialysis initiation

Figure S1-B

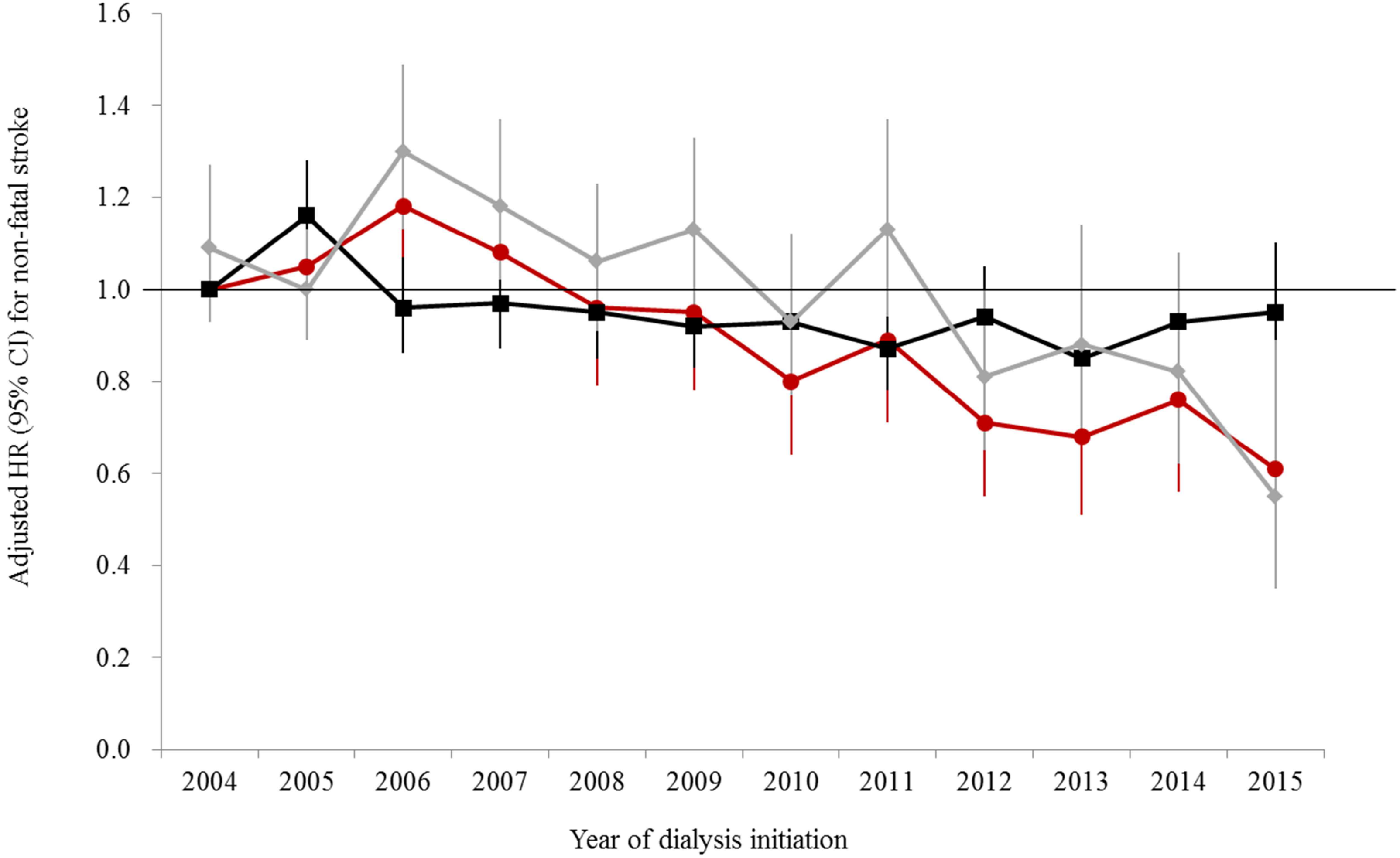

Adjusted HR (95% CI) for non-fatal stroke
1.6
1.4
1.2
1.0
0.8
0.6
0.4
0.2
0.0
2004
2005
2006
2007
2008
2009
2010
2011
2012
2013
2014
2015
Year of dialysis initiation

Figure S1-C

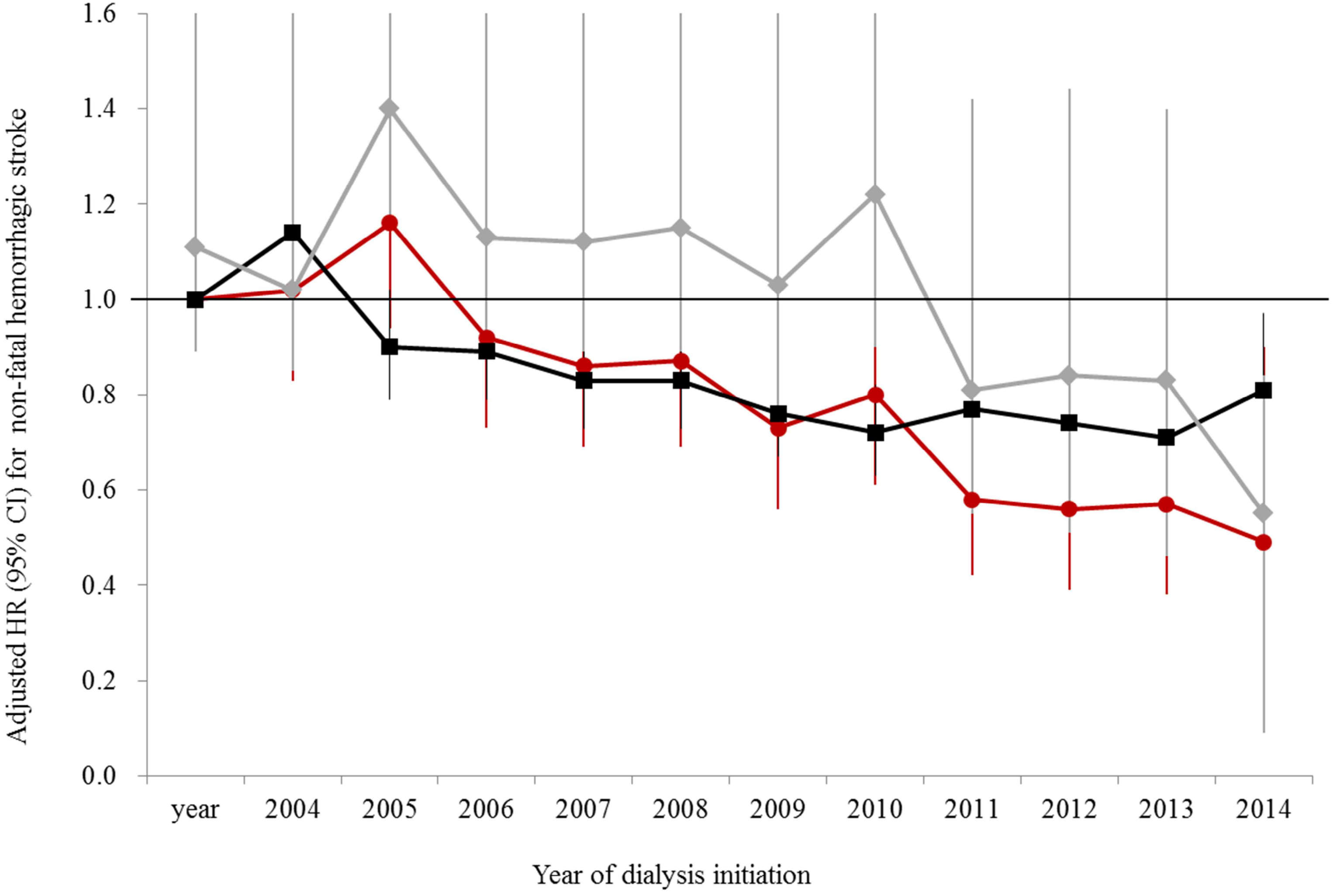
1.6
1.4
1.2
1.0
0.8
0.6
0.4
0.2
0.0
Adjusted HR (95% CI) for non-fatal hemorrhagic stroke
year
2004
2005
2006
2007
2008
2009
2010
2011
2012
2013
2014
Year of dialysis initiation

Figure S1-D

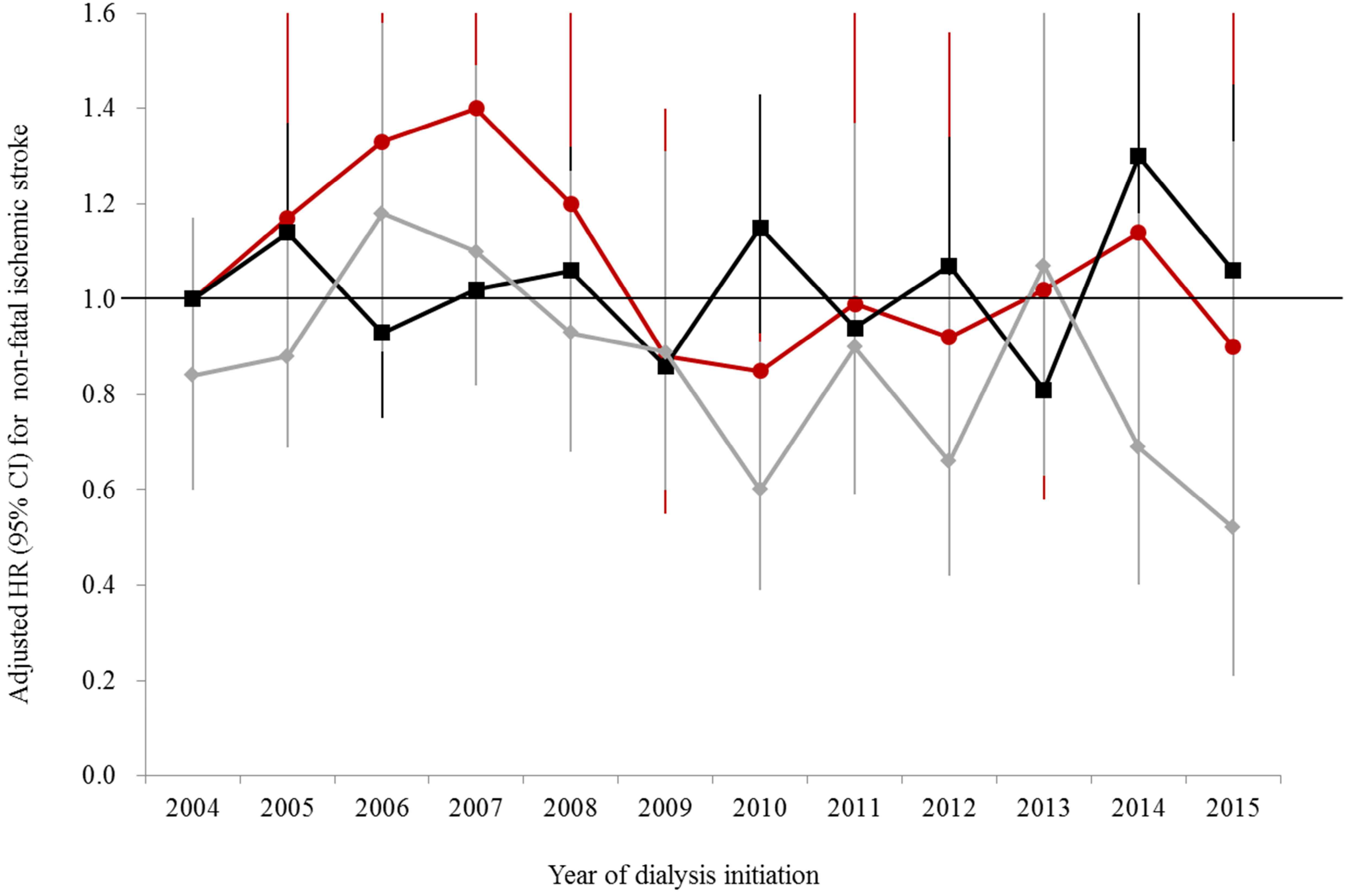

1.6
1.4
1.2
1.0
0.8
0.6
0.4
0.2
0.0
Adjusted HR (95% CI) for non-fatal ischemic stroke
2004
2005
2006
2007
2008
2009
2010
2011
2012
2013
2014
2015
Year of dialysis initiation
